# The Relational Nature of Gender, the Pervasiveness of Heteronormative Sexual Scripts, and the Impact on Sexual Pleasure

**DOI:** 10.1007/s10508-023-02558-x

**Published:** 2023-02-21

**Authors:** Penny Harvey, Erielle Jones, Daniel Copulsky

**Affiliations:** 1 Human Sexuality Department, California Institute of Integral Studies, 1453 Mission St., San Francisco, CA 94103 USA; 2 Department of Sociology, University of Illinois Chicago, Chicago, IL USA; 3 Psychology Department, University of California, Santa Cruz, Santa Cruz, CA USA

**Keywords:** Scripts, Pleasure, Cisnormativity, Heteronormativity, Gender

## Abstract

This study examined how gender shapes sexual interactions and pleasure outcomes. We highlight varying expectations people have in regard to sex by combining questions about orgasm frequency and sexual pleasure. Our analysis was driven from a sample of 907 survey responses from cis women, cis men, trans women, trans men, non-binary, and intersex millennial respondents, 324 of which had gender-diverse sexual histories. The findings built upon previous literature about the orgasm gap by including those with underrepresented gender identities and expanding our conceptualization of gender’s role in the gap beyond gender identity. Qualitative results indicated that individuals change their behavior based on their partner’s gender and follow strong gendered scripts. Participants also relied upon heteronormative scripts and cis normative roles to set their interactions for the sexual encounter. Our findings support previous research on how gender identity impacts pleasure outcomes and has implications for how we might make gender progress in the arena of sexuality.

## Introduction

Despite advancements in gender equality in many aspects of our social lives, our sexual interactions remain deeply gendered. Why does gender affect a person’s likelihood to orgasm in a sexual encounter? Research has uncovered an “orgasm gap,” in which women have orgasms less frequently during sexual interactions than men (Bryan, 2001; Darling & Davidson, 1986; Fahs & Swank, 2013; Muehlenhard & Shippee, 2010; Opperman et al., 2014; Wiederman, 1997). Scholarship has moved from biological explanations to examining the influence that sociocultural factors such as gender norms, power dynamics, and heteronormativity have on the persistence of the orgasm gap (Fahs & Swank, 2013; Narvaja, 2016). What remains largely unexamined are the ways in which orgasm outcomes change according to the gender of one's sexual partner.

Millennials are assumed to have a more progressive understanding of both gender and sexuality but scholarship on sexual behaviors and outcomes does not reflect that (Milkman, 2017; Wade, 2017; Wilcox, 2011). For example, studies primarily address the orgasm gap from cisgender (cis) perspectives, and commonly those in monosexual pairings. Studies focusing on the orgasm gap are commonly exclusive to those in same or opposite-sex pairings and show that cis men have high rates of orgasm (Allen & Carmody, 2012; Frederick et al., 2018; Narvaja, 2016) particularly when partnered with cis women, but cis women orgasm rates increase when partnered with cis women (Blair et al., 2018). Heterosexual cis men and cis women have the greatest orgasm disparity, due in part to ending sex at the moment of men’s ejaculation, yet this does not fully account for the differences (Allen & Carmody, 2012; Narvaja, 2016). Moreover, previous studies show that while cis men tend to define pleasure in terms of orgasm, gender minorities (e.g., trans, non-binary and intersex people) do not define sexual satisfaction as orgasm alone has more expansive criteria for determining sexual satisfaction (Halwani, 2018; Metz & McCarthy, 2007; Smith, 2007; Williams et al., 2016). These studies help us look comparatively at cis gender persons and at the differences between heterosexual, lesbian, and gay partnerships. However, they do not expand our understanding of how cis gender outcomes compare to trans, non-binary, and intersex, or how gender structures sexual interactions and influences pleasure beyond gender identity. Moreover, previous studies show that while cis men tend to define pleasure in terms of orgasm, gender minorities do not define sexual satisfaction as orgasm alone have more expansive criteria for determining sexual satisfaction (Halwani, 2018; Metz & McCarthy, 2007; Smith, 2007; Williams et al., 2016).

Sexual relations are a key place where gender identity and gender make-up (the elements of gender that form someone’s overall gender; Harvey, 2020) may be troubled and affirmed. Previous research shows that gender minorities and Lesbian/Gay/Bisexual + (LGB) individuals often rely more heavily upon verbal communication to navigate gender expectations, whereas cisgender individuals in heterosexual partnerships commonly use nonverbal cues (Jamieson, 1999; Julien et al., 2003; Rubinsky & Hosek, 2020). We draw on the theories of doing gender (the process of performing or enacting one’s gender) and sexual scripts (the sexual norms and expectations set by society) to examine how respondents bridge the gap in communication (Gagnon & Simon, 1987; West & Zimmerman 1987). Qualitative responses illuminate, beyond the quantitative findings of this project, the relationality of gender as respondents discuss how their level of communication and sexual behavior differs according to their partner’s gender.

To examine these relationships, we utilize a mixed methods survey data set sampling folks across all gender identity groups. We draw from both quantitative and qualitative responses using an integrative approach to better understand the impact of gender on sexual pleasure outcomes, and the meaning of gender in sexual interactions. Our findings show that outcomes for sexual pleasure are impacted by individuals’ own identities and gendered experiences in conjunction with the gender/sex of their partners (van Anders, 2015). In many instances, participants point to the ways in which heteronormativity and cisnormativity are reproduced in sexual relationships, including those with same-gender, transgender, and non-binary people, as individuals take on gendered roles depending on the gender/sex and gender expression of their partners (Bauer et al., 2009; Lindley et al., 2022; Ward & Schneider, 2009).

We argue that sex is an interaction that is deeply affected by gender norms, expectations, and scripts (Tiefer, 2004). By surveying individuals who have sex with partners of various genders as opposed to those with monosexual desire, we could better theorize the extent to which gender expectations and socialization might contribute to the orgasm gap. Moreover, we were able to understand how orgasm rates compare for transgender, non-binary, and intersex people and the level of significance orgasm has in their interactions. To eliminate certain biological arguments that people with vulvas are more likely to experience sexual dysfunction, we eliminated people who had medical conditions that affected sex, and people who had not ever orgasmed from the sample (Cacchioni, 2007; Fahs, 2014). Despite this approach, our findings on cis men and women were consistent with existing literature, demonstrating that (inferior) biology is not explanatory for sexual inequalities. We also found that gender is relational: The gendered position our respondents took was affected by the assumed gender (role) of their partner.

### Doing Gender and Receiving Gender

Doing gender asserts that gender performance allows the presumption that people are making their sex or more specifically, their genitalia, knowable which facilitates heterosexual unions (West & Zimmerman, 1987). Studies by queer scholars have shown that people’s assumptions about genitalia and not doing gender correctly have serious consequences, including termination of intimate relationships, public outing and ridicule, and the murder of trans women by their cis men sexual partners (Bogle, 2008; Lamont, 2014; Lindley et al., 2022; Muñoz-Laboy et al., 2017; Schilt & Westbrook, 2009; Zamantakis, 2020). Along with assuming sex, doing gender works to enforce a gender hierarchy. Gendered interactions work to communicate the expectation that men are dominant and women are submissive (McCreary & Rhodes, 2001; West & Zimmerman, 1987, 2009), and this expectation contributes to vast inequalities in sexual relations between cis men and women (Bogle, 2008; Jackson & Scott, 2002; Lamont, 2014). Queer feminist scholars argue that the institutionalization of heterosexuality gives the appearance that there are biological and social opposites, thus naturalizing the link between sex categorization and gender hierarchy (Butler, 2002; Ingraham, 1994; Schilt & Westbrook, 2009).

The repetitive practice of doing gender solidifies gender norms and provides a framework for how we should conduct ourselves in various social situations (Ridgeway, 2011). While there are multiple understandings of gender norms, we follow the definition offered by Keleher and Franklin (2008): “powerful, pervasive values and attitudes, about gender-based social roles and behaviors that are deeply embedded in social structures,” ensuring “the maintenance of social order, punishing or sanctioning deviance from those norms” (p. 43). Though the norms associated with a gender identity have social and cultural recognition, they are not fixed and universal. Internal/interpersonal factors, socialization, economic, cultural and social capital, structural factors, cultural influences, and education can all impact how someone interprets and follows social norms and thus “does gender” (De Meyer et al., 2017; Lundgren et al., 2019; Tolman et al., 2003). Implicit in our understanding of the doing of gender is the link between gender norms and underlying ideologies of power (Keleher & Franklin, 2008; Lundgren et al., 2019; Marcus & Harper, 2014). Even when folks do not live traditional or normative manifestations of gender, they understand the norms and expectations of gender and how masculinity and femininity should be performed (Lindley et al., 2022). More importantly, they are also aware of the risks associated with living outside the norms, and they are aptly able to engage in behaviors in a way that calculates the risks associated with their gender performance, and understand how their behavior will or will not be classified (Kattari et al., 2021; Lindley et al., 2022; West & Zimmerman, 1987; Zamantakis, 2020).

Scholars have questioned the impact that changing/improving structural gender equality has had on gender norms in the context of relationships. Despite changing attitudes and structural progress, actions perpetuating gender inequality in relationships remain (Eaton & Rose, 2011; Lamont, 2014). Individuals know that they will be held accountable to their gender performance if they do not live up to normative conceptions of femininity or masculinity (West & Zimmerman, 1987). After violating gendered social norms, many people experience shame or guilt, even when they strongly believe in gender equality in relationships (Lever et al., 2015).

Though there are many factors that impact an individual’s adoption and/or interpretation of gendered norms and script, safety and culture remain highly influential. Their assessment in turn impacts how they do gender and means they may do gender differently across social situations. We aim to assess how someone does gender in relationships and specifically sexual interactions, and the impact of those choices on sexual pleasure and satisfaction.

### Heteronormative Sexual Scripts and the Impact on Pleasure

Jackson (2006) argued that heterosexuality is a key site of the intersection between gender and sexuality where we can see gender inequalities and the marginalization of other sexualities. Heterosexuality is often treated as the standard by which non-heterosexual encounters are measured because heterosexuality dominates representation of sexual interaction. Despite their explicit rejection of heteronormative scripts, there is evidence that LGBTQ sexual and romantic interactions often reify heteronormativity; the reliance upon heterosexuality as the normative standard for sexual practice, gender relations, and ways of life (Harvey, 2020; Jackson, 2006; Jamieson, 1999; Muñoz-Laboy et al., 2017; Spišák, 2017). They also challenge heteronormativity by regularly communicating, evaluating, and discussing consent (Harvey, 2020; Muñoz-Laboy et al., 2017; Spišák, 2017). Regardless of evidence of some change and subversion in queer relationships, heteronormativity still seems to have a big impact on sexual relationships.

Gagnon and Simon (1987) stated that scripts operate at three levels to moderate the way that we interact with other people. While we learn the general expectations from sexual cultural scripts depicted in media and popular discourse, we develop interpersonal scripts from our own experiences and use our intrapsychic scripts to rehearse interactions and determine our desires. The language of scripts is widely used to understand heterosexual relationships and contributes to our knowledge about the influence of heteronormative scripts in sexual encounters (Sakaluk et al., 2014; Sanchez et al., 2012). However, Wiederman (2015) notes that few if any studies centering trans folks use scripts theory and only a small number of studies center non-heterosexual populations (Lindley et al., 2022).

The underuse of script theory when studying trans and non-heterosexual populations may be due to the aspect of sexual homeostasis embedded in the theory: the idea that after years of sexual encounters, one maintains the script that ensures adequate performance and pleasure (Wiederman, 2015). This presumes a relative stability of gender and sexuality; however, Jackson (2006) reminds us that sexual life is variable because our gender and sexuality are renegotiated throughout our lives. Thus, the fluidity of gender and sexuality could mean that people with gender-diverse sexual histories have many interpersonal scripts and intrapsychic scripts to guide their interactions. As people define their gender, their intrapsychic scripts (desires) may change. For example, trans men and women often report that their sexual desires changed as they renegotiated their relationship with their bodies and held themselves accountable to their new gender (Brown, 2010; Gieles et al., 2022; Pfeffer, 2008; Schrock & Reid, 2006). Cis women partnered with trans women also alter their sexual behavior, relying upon interpersonal scripts from previous relationships with men (Brown, 2010).

Drawing on insights from doing gender, heteronormativity, and scripts theory, we aim to understand how people alter their sexual performance and expectations in accordance with their sexual partner’s gender. Doing gender suggests that people rely heavily on their understanding of their own masculinity, femininity, and sexuality to navigate sexual encounters. Scripts theory tells us that people draw on popular representation, education (formal and informal), personal experience, and desires. Both theories refer to heteronormativity to describe the interwoven nature of gender and sexuality, but this application is often restricted to cis heterosexual, trans heterosexual, and same-sex relationships. Does heteronormativity serve as the referent in other types of relationships? Does performance reflect accountability to one’s own gender or does performance shift to affirm the partner’s gender?

Several studies have found that heterosexual women are less likely to experience orgasms than heterosexual men, lesbian women, and gay men, with heterosexual men being the most likely to orgasm in a sexual encounter (Allen & Carmody, 2012; Frederick et al., 2018; Narvaja, 2016). Scholars often attribute the “orgasm gap” to the expectation that heterosexual encounters end after the man orgasms (Allen & Carmody, 2012; Narvaja, 2016). Yet, all of these groups report similar levels of sexual satisfaction (Blair et al., 2018). These studies suggest that sexual scripts set an expectation that women will have sex without achieving an orgasm, which helps women reconcile the infrequency of orgasms. We question how this compares to experiences of bisexual, queer, pan, non-binary, and trans folks, who have yet to be included in this research. Existing research on trans sex is limited (Bradford & Spencer, 2020). Studies inclusive of trans participants looking at gender inequality in sexual relationships are even more limited, and thus, more research needs to be done that is inclusive of a plurality of gender identities.

Gender is performed in sexual interactions, deeply affecting the expectation of pleasure, as well as the outcome, in many monosexual, cis gender partnerships. As detailed in the above literature, current research explores how sexual interactions are gendered for cis folks and most commonly focuses on monosexual pairings. We expand on existing research to investigate the gendered patterns as they relate to pleasure and expectations beyond the cis/het binary.

## Method

Stemming from “The Pleasure Study'' data set (Harvey, 2020–2021), this paper employs an integrative mixed-methods approach (Creswell et al., 2011). We drew from qualitative questions on sexual interactions answered by participants who had had sexual interactions with people of more than one gender (*N *= 324) as well as quantitative questions on participants’ gender, their last sex partner’s gender, and sexual pleasure that drew from the data set as a whole (*N* = 907). We integrated these findings using a concurrent approach to build a well-rounded, in-depth picture of how the gender of both partners shapes sexual interactions (Teddlie & Tashakkori, 2006).

### Participants

Our sample consisted of 11.4% cis men, 46% cis women, 7.5% trans men, 4.0% trans women, 24.5% non-binary people, 5.0% intersex people, and 1.6% who selected other gender. A total of 27.0% of our sample was straight and 73.0% identified as LGBQ. Our racial/ethnic makeup, multi-select self-identified was 0.5% Native American, 3.9% Asian, 7.2% Black, 4.6, Latinx, 0.2% Jewish, 0.4% Middle Eastern, 62.0% white, 1.9% other race, and 18.5% multi-race/ethnicity. 40.3% of the sample were self-identified as working class, 49.4% middle class, and 10.3% upper class.

### Procedure

Data for this study were collected in two waves. The first wave was collected between March and April of 2020, by Author 1 for their dissertation and follow-up research. The second wave was collected between March and May 2021 by a team of graduate research assistants and used purposive sampling to expand the gender diversity in the data set. While cis/het men and women were not purposely recruited in the second wave (flyers called for trans men, trans women, non-binary, and intersex respondents), they were not excluded from response as the participation criteria remained the same in both waves. Study participants were recruited through online spaces, including social media and organization listservs and newsletters. When recruiting in specialized spaces, such as LGBTQ groups, recruitment materials were adapted to specify a need for group members. Recruitment materials directed potential participants to the central study website where they could click the survey link and/or provide their email for future research. Potential participants were incentivized with a potential to win a $25 gift card in a raffle and were given additional entries into the raffle for recruiting others. To be eligible for the survey, participants had to be between 21 and 38 (Wave 1) and 22 and 39 (Wave 2), and have had sex.

Georgia State University Institutional Review Board approved Wave 1 of the study, and the California Institute of Integral Studies Human Research Review Committee approved Wave 2 of the study. The survey began with an informed consent form and eligibility criteria confirmation to which “I agree” was selected before further access to the survey was provided. No identifiable information was collected with the data; upon completion, the survey redirected to a separate form for respondents to provide an email address and/or recruitment code to be entered into the raffle.

The survey received 638 valid responses in Wave 1 and 545 in Wave 2, totaling 1183 responses. The survey had 69 total questions and took an average of 17 min to complete. Question blocks included sections on gender, sexual history, sexual partners, last sexual interaction, last sexual partner’s gender, sexual empowerment, gender dysphoria, and demographics. The data were coded and cleaned by the first author and a team of graduate students and early career scholars as part of a summer fellowship at the California Institute of Integral Studies.

### Measures

#### Gender

Participants were asked which gender identity label they most commonly use, checking all that apply from 9 options: agender/genderless, intersex, man, non-binary, trans, trans man, trans woman, woman, and other. Participants were also asked which sex or sex markers apply best for them, checking all that apply from six options: female, intersex, male, no sex, trans, and other. Based on these questions, participants were categorized as cis man, cis woman, trans man, trans woman, non-binary, other gender, or intersex. Anyone who selected intersex from either question was categorized as intersex. Participants were categorized as a trans man if they selected trans man, both man and female, or either trans option along with either man or male. Likewise, participants were categorized as a trans woman if they selected trans woman, both woman and male, or either trans option along with woman or female. (Participants could be categorized as a trans man or trans woman even if they also selected additional options beyond those mentioned above, but were not categorized if they met the criteria for both trans man and trans woman.) Remaining participants were categorized as non-binary if they selected the agender/genderless or non-binary options, we did not distinguish between non-binary folks who selected trans markers and those who did not. Participants were categorized as cis women if they selected only woman and female (those who selected these along with “other” were also included). Similarly, participants were categorized as cis men if they selected only man and male (those who selected these along with “other” were also included). We did this as the data did not have a cis option, so our “best guess” at accurate categorization was treating those who only selected man or woman and the corresponding sex (even if they selected other) as cis. The authors made this coding choice as many trans men and trans women were also selecting “man,” “woman,” “non-binary,” and “other” in addition to the trans man label. We wanted to ensure that the front end of the survey was as inclusive as possible while having to categorize folks on the back end for statistical analysis. The sample size was not large enough to separate respondents into multiple selection categories such as “trans man, non-binary” and “trans man binary.” Despite this limitation, we argue that the data collected still provide important information about the gender identity groups collected.

#### Orgasm

Participants were also asked about their orgasm experiences the last time they had sex, with the question “did you orgasm?” Three response choices were offered: yes, “maybe/not sure,” and no.

#### Communication

We also measured communication with questions extracted from the Sexual Assertiveness Questionnaire (Loshek & Terrel, 2015). We extracted any questions related to respondent communication as detailed in their instrument. They were then compiled and averaged to provide continuous communication “scores.” These items were scored with a slider bar on an 8-point scale from 1 (strongly agree) to 8 (strongly disagree).

#### Gender-Diverse Sexual Partners

Some of our analysis was limited to those participants who had sexual experience with multiple genders, to which we asked quantitative and qualitative questions. This subsample is defined as those who said it was true that they “have had sex with people of more than one gender.” These participants were asked the gender identity of their last sexual partner, again checking all that apply from the same 9 options as the question on participant gender identity and categorized following the same previously detailed approach. Participants were also asked to specify how masculine and feminine they consider their last sexual partner to be, each measured on four scales: “I feel as though they are,” “they look as though they are,” “they do most things in the manner typical of someone who is,” and “their interests are mostly those of a person who is.” These responses were entered with a slider bar on a 1–5 scale, with 1 marked “not at all,” 3 marked “somewhat,” and 5 marked “very” (see Appendix for full version). They were then averaged to create their individual femininity and masculinity gender make-up (Harvey, 2020). The Cronbach alpha was 0.81 which indicates a high level of internal consistency for our scale.

This group was then asked three questions. They were first asked whether their “expectations of sex (and the sexual interaction) changes based upon the gender of my partner(s).” Participants selected from three options: true, false, or “I had no gender expectations for them.” Then participants were asked whether “the way in which I have sex or act during sex changes with different gendered partners?” True and false options were offered here. Participants were last offered an open write-in field where they could “explain how / if the way in which you interact with someone during sex is affected by their gender.”

### Analytic Plan

We took an integrated approach to analyze the data. The quantitative data were analyzed using SPSS 26. Descriptive measures were used to assess frequencies, then a series of crosstabs and chi-squared to assess association. Alpha values were set at *p* < 0.05 and considered statistically significant.

During the survey, we also elicited qualitative data by asking respondents how their expectations changed according to their partner’s gender. We used a thematic approach to analyze qualitative responses (Charmaz, 2014; Corbin & Strauss, 1990; Saldaña, 2021).

The qualitative phase was conducted with thematic joint coding of the qualitative response questions, as detailed above. The first and second authors first engaged in open coding, clustering responses together that appeared to touch upon the same themes. Then using focused coding, we noted responses that hit upon many different themes by extracting sections of the quote and commented on additional areas that fit. The first and second authors resolved disagreement about the compatibility of responses by creating subthemes to unearth the tensions in seemingly similar responses. For example, in the initial coding stage a cluster emerged that had too many responses to make a coherent argument. After much deliberation, we realized that there was a difference between gendered expectations that reflected cisnormativity and those reflected heteronormative scripts.

Within the gendered expectations, we further noted that some respondents spoke only of their expectations of their partner while other respondents focused on the expectations imposed upon them by their partners. As will be shown in our findings, such discoveries prompted the second author to identify respondent gender and last partner’s gender to understand the relationship between gender and sexual expectation. In the analysis, both partner’s gender and respondent’s gender contributed to divergent responses. In the findings section, respondent gender is denoted in parentheses next to their assigned pseudonym (I = intersex, TM = trans man, TW = trans woman, CW = cis woman, CM = cis man, N = non-binary, O = other).

After deciding that scripts theory best supported most of the data, the second author revised codes to align with the scripts framework. For example, heteronormativity is well explored in scripts theory and the reliance upon heteronormative scripts often stands in the way of partner communication. Heteronormative scripts also assume binary feminine/masculine or a dominant/passive relationship between sexual partners which is a recurrent theme. Finally, a number of our respondents spoke about sexual triggers or violent histories that informed their expectations; these responses spoke to the detrimental nature of scripts. To keep our argument succinct, we dropped initial themes that were less common or did not speak to script theory during the selective coding stage of the coding process. All authors reviewed the data and agreed upon the themes and analysis. The qualitative data included in the analysis list the respondent’s gender and pseudonym we assigned them.

Integrative work was then done to connect the qualitative and quantitative findings. A concurrent approach was then employed to understand the quantitative and qualitative results in tandem (Jang et al., 2008; Teddlie & Tashakkori, 2006). Both sets of data were initially analyzed independently as detailed above. We then met to review both sets of data and findings together. We asked how did the results complement and contradict each other, how did they provide context, and in what ways were the respondents' stories deepened and complicated by the integration of the findings? Results from both sets of data were synthesized and connected and formed the sections of the paper during the writing up of the results.

## Results

### The Effect of the Sexual Partners’ Gender on Pleasure

In order to understand the impact of gender identity on sexual interactions, we asked respondents about their last sexual interaction. A total of 899 of 907 respondents who had had sex in the past 6 months reported the gender identity of their last sexual partner (see Table 1). The largest sexual partner makeup consisted of cis man/cis woman pairings which accounted for 43.8% of responses, followed by non-binary/cis man partnerships at 11.4%, non-binary/cis woman pairs made up 7.0% of the sample, 6.9% were cis women/cis woman, 4.9% were non-binary/non-binary, 4.1% were cis man/cis man, and 4.1% were cis woman/trans man.^1^ Other pairings made up less than 2% of responses.

**Table 1:** Partner distribution frequency

Partner distributions	Frequency	Percent
Cis woman/Cis man	325	36.2
Non-binary/Cis man	99	11.0
Cis man/Cis woman	68	7.6
Cis woman/Cis woman	62	6.9
Non-binary/Cis woman	45	5.0
Non-binary/Non-binary	44	4.9
Trans man/Cis woman	32	3.6
Cis man/Cis man	29	3.2
Trans man/Cis man	19	2.1
Intersex/Cis man	19	2.1
Cis woman/Non-binary	18	2.0
Trans woman/Cis man	15	1.7
Non-binary/Trans woman and intersex/Cis woman	13	1.4
Non-binary/Trans man	11	1.2
Trans woman/Cis woman	8	0.9
Trans man/Non-binary	7	0.8
Non-binary/Other and other/Cis woman	6	0.7
Trans man/Trans woman, Trans woman/Non-binary, Intersex/Trans woman and Intersex/Non-binary	5	0.6
Cis Man/Non-binary, Cis woman/Other	4	0.4
Cis woman/Trans man, Trans Man/Trans man, Trans woman/Trans man, Trans woman/Trans woman, other/Cis man and other/Non-binary	3	0.3
Cis man/Trans man, Trans man/Intersex, Trans woman/Other, Trans woman/Other, Non-binary/Intersex, intersex/Other, other/Trans woman, Cis woman/Intersex, Intersex/Trans man	2	0.2
Total	899	100

Where multiple pairings are listed, the frequency and percentages represent the number for each pairing, not the pairings collectively

**Fig. 1:**
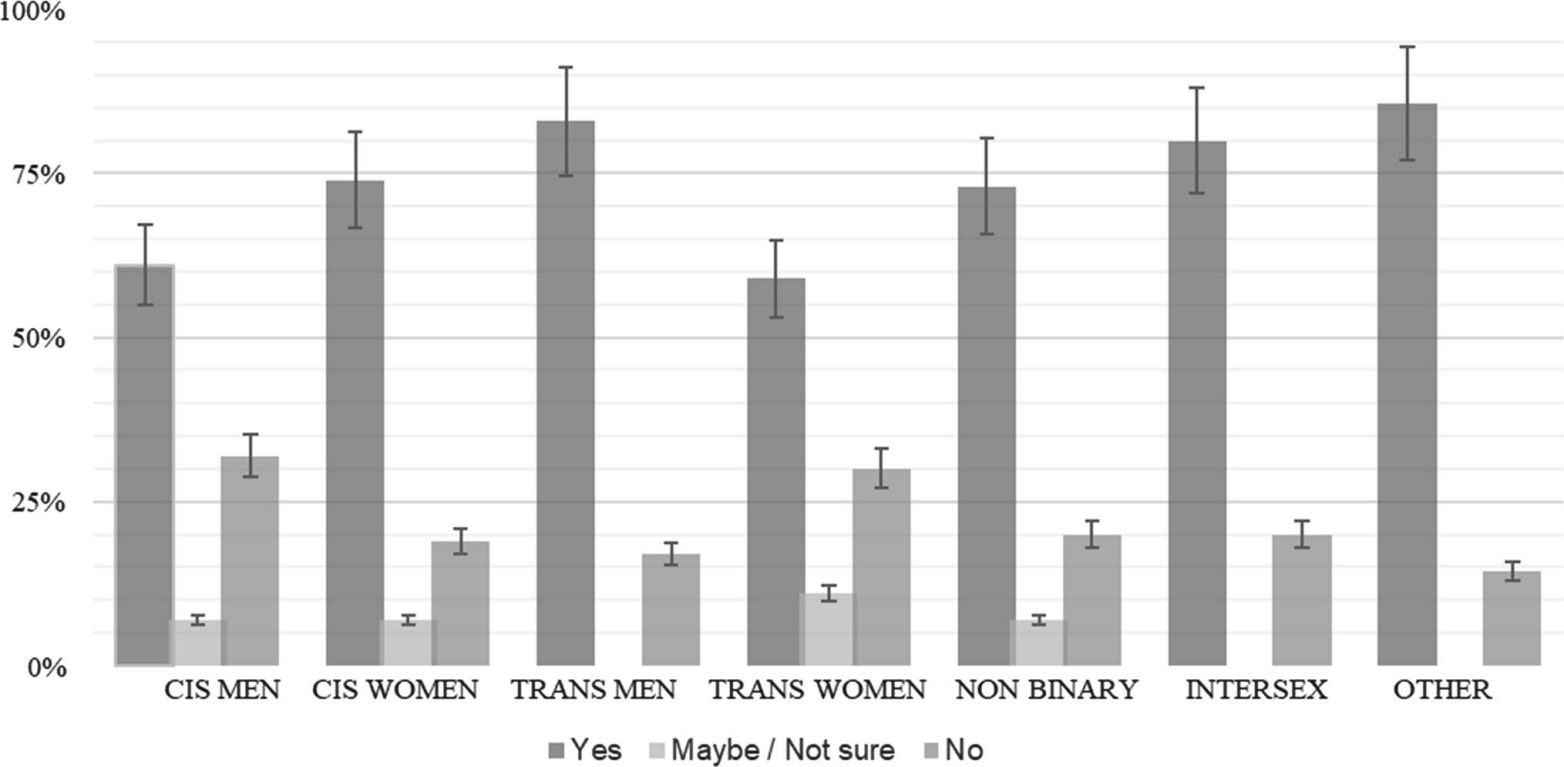
Orgasm at last sexual interaction and partner gender ID. All were statistically significant at the 0.01 level *χ*^2^(1) = 38.95

**Fig. 2:**
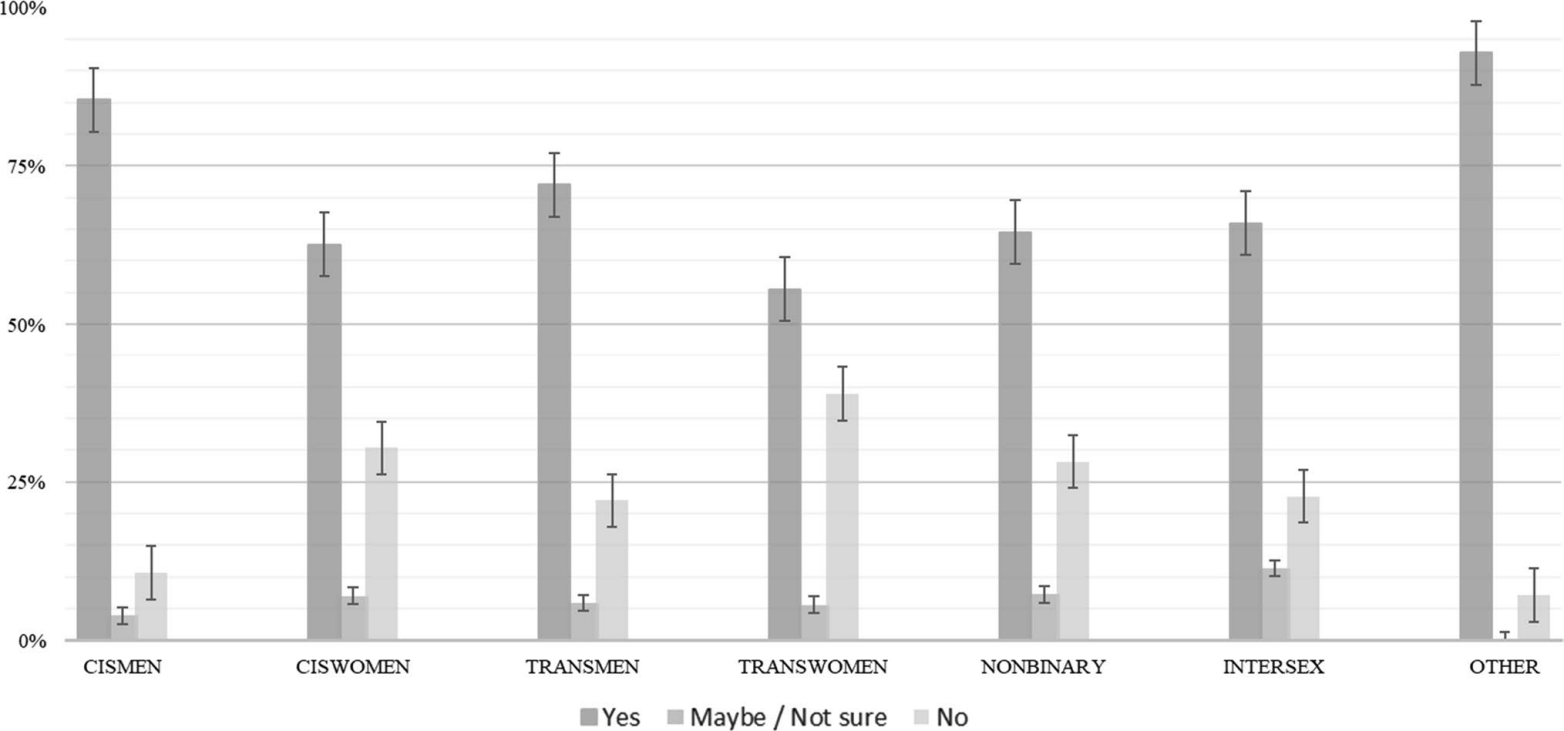
Orgasm at last sexual interaction and respondent gender ID. All were statistically significant at the 0.01 level *χ*^2^(1) = 29.94

Figure 1 shows significant differences (chi-squared = 0.01) for the frequency of orgasm at the last sexual encounter based on the sexual partner’s gender. Individuals partnered with cis men (CM) and trans women (TW) had the lowest orgasm frequency and those partnered with trans men (TM) or other (O) had the highest orgasm frequency. Individuals with non-binary (N) partners or cis women (CW) as last sexual partners had similar outcomes. Cis men were most likely to orgasm, regardless of their partner’s gender, and had the highest outcomes when their last reported partner was a cis woman (see Fig. 2). Cis women with cis men as their last sexual partner were the least likely to orgasm. Respondents were most likely to orgasm if their last partner was identified as a cis woman, trans man, non-binary, or intersex (I). Those who reported cis men as their last sexual partner were generally less likely to report orgasm.

To find out more, a follow-up question was asked to respondents who had had sex with partners across more than one gender identity group. When asked whether they agree with the statement “My expectations of sex (and the sexual interaction) changes based on the gender of my partner(s),” Table 2 shows that 52.0% agreed that their expectation of sex and the sexual interaction changed based upon the gender of their partner and 35.7% stated that they did not have any gender expectations. Further, when asked whether they agree with the statement “The way I have sex or act during sex changes with different gendered partners,” 62.5% respondents agreed with the statement. Very few respondents stated that gender did not alter the way they had sex and those who did were primarily cis men. Moreover, those who did not indicate a change in gendered expectations or had no gendered expectations at all reported higher incidences of orgasm during their last sexual encounter. Therefore, by pulling these findings together in context we can confidently say that the sexual partner’s gender impacts both the expectations and outcomes (in this case orgasm) of a sexual interaction. To understand more about the impact of gender, we asked respondents to explain how the expectations changed because of their partner's gender.

**Table 2:** Sexual interaction expectations and actions based on gender

	Sex acts change based on partner’s gender	Expectations of sex changed based on partner’s gender
True	63% (349)	52% (290)
False	37% (205)	12% (67)
I had no expectations for them based upon gender	N/A	36% (199)
Total	100% (554)	100% (556)

When asked to expand on how expectations of a sexual interaction change according to their partner’s gender, 320/324 respondents provided qualitative responses. We found that the qualitative responses indicate a reliance on genitalia to guide sexual acts and dependence on sexual scripts in lieu of communication. This finding fits with West and Zimmerman's (1987) assertion that gendered assumptions are aligned with genitalia and produced heteronormative, phallic centered scripts. We find this reflects heteronormative scripts typical in orgasm gap and sexual pleasure research. Unique to our findings is that this occurrence is happening with non-binary, queer, trans, and other respondents who have potentially rejected heteronormative and/or binary ways of existing in the social world. Therefore, we highlight the pervasive nature of heteropatriarchy and its influence on our most intimate sexual interactions. Moreover, our findings indicate that heteronormativity and binary/conventional gendered meanings are reinforced through unintentional and often unconscious social processes.

### Cultural Expectations of the Body

Due to the aforementioned findings, having a cis man partner or trans woman partner led to the lowest occurrences of orgasm at last sexual interaction, we questioned the role of anatomy and socialization in sexual interactions. We posited that socialized gender, rather than current gender, may be impacting the scripts and thus expectations in a sexual interaction. However, as the following quotes demonstrate, respondents frequently assessed the possibilities of their encounter based on their partner’s genitals. The inattention to socialized gender in these responses suggest a strong relationship between cisnormativity and salience of heterosexual scripts.

The cultural expectation of sex with a man was heavily commented on or used as the referent for assessing other sexual interactions. These responses referred to men and women without much distinction between trans and cis partners, and coherence between sex and gender was implied through the interchange between cultural and biological explanations. For example, Georgia (CW) noted that it is harder to negotiate pleasure or have an orgasm with men partners which she attributes to their “biological limitations,” “the gendered expectation that they will not continue after they’ve “finished,” and that she must reciprocate all of her requests during sex.

Alex (N) also draws on cultural expectations to explain their divergent experiences with men and women:

I assume that when having sex with men they will want oral sex and/or vaginal penetration and that they expect to climax and are often not fulfilled if they don’t climax during sex. My expectation of women is much more open ended, and I don’t expect anything in particular. I also don’t assume that a woman will necessarily expect to climax and/or be unfulfilled if they don’t experience climax.

Stevie (N) similarly stated “Sexually, men tend to be more goal oriented, in that it’s pretty much just about the orgasm. Women tend to be more about the experience.” These accounts show not only the binary way people still think about sex and gendered sexual expectations but also how much sexual expectations for cis men and women differ. Importantly, Alex (N) and Stevie (N) do not make a distinction between trans women and cis women in their responses but note that women do not expect an orgasm.

Quantitative data show that trans women in our study had orgasms at the lowest frequency of the gender identity groups. Unlike cis women who thought of differences in terms of orgasm frequency, trans women did not discuss orgasm frequency at all. Trans women primarily discussed their ability to top their partner with declarations such as “I can’t top men as easily.” Priscilla (TW) focused on anatomical differences by referring to her partner’s sex categorization as opposed to gender:

While having sex with a male, I tend to take on the role of a female. While having sex with a female, I tend to take on the role of a male.

Priscilla’s (TW) use of sexed language rather than gendered language also creates a distinction between sex and gender, but indicates that genitals signal the kind of sex that will occur. Cis men and intersex respondents similarly noted that the only difference was reference to the type of penetrative sex that would ensue. For example, Mark (CM) said:

It is unaffected by gender, but is affected by what genitalia they have - obviously cannot have vaginal penetration without a vagina. Other than that, it is the same.

Frankie (I) also said:

Mostly it’s in how we actually have penetrative sex, and the length of time. Sex is shorter with men or partners with male appearing parts. Otherwise it’s not as different.

Therefore, even when gender is deemed unimportant, biological sex or genitalia guide role assignment based on heteronormative scripts. These scripts center penetrative sex, trivialize cis women’s pleasure, and end with ejaculation. Many of our participants indicated “progressive” approaches to the understanding of gender and sexual interactions, yet we still found strong gendered associations and patterns in our data about sexual interactions.

Even those who quantitatively reported that gender did not impact their expectations or actions in a sexual interaction, many still responded in ways that reflect gendered behavior. As indicated by Mark’s quote, sometimes changes were related to anatomy. Some respondents noted that differences were also related to conduct and language:

George (CM): All that changes is how I speak to them. A more fem person/woman would be called beautiful and such. A masc person/guy would be called handsome and such.

Despite many gendered ways of discussing expectations in sexual interactions some folks remained ungendered in their responses framing their expectations in terms of sexual energy or simply personal preference. Therefore, the finding that those with no gender expectations or whose expectations of gender did not change based upon their partner’s gender were more likely to have experienced orgasm during their last sexual interaction compared to those with gendered expectations is important to show the ways in which gender can impact sexual satisfaction.

### Orgasm as a Goal

Though orgasm is not the only measure of sexual satisfaction, or good sex, it remains a key aspect of sexual interactions. Participants across all gender categories said that women’s (presumably cis) satisfaction did not depend upon achieving an orgasm (see Table 3). In their qualitative responses, they also described women as more attentive and giving, whereas men were goal-oriented or focused on achieving their own orgasm. Blair et al. (2018) argue that heteronormative scripts best explain why women in heterosexual relationships have worse outcomes than lesbians but genderqueer respondents in our study maintained that women do not need to orgasm. Our findings suggest that non-heterosexual relationships share the expectation that women do not need an orgasm to experience pleasure to the contrary of cis women’s actual desires. Only 13% of our cis women sample asserted that orgasm was not a goal during sex, whereas 52% were unsure, and 35% said orgasm was a goal. So why is there such a difference in the perceived expectations of women’s sexual goals and women’s reported sexual goals, even in same-gender partnerships? More research is certainly needed to understand whether other outcomes follow such gendered patterns and whether overall sexual satisfaction reports show a gender gap.

**Table 3:** Orgasm goal by gender identity

		Yes	No	Unsure	Total
Gender ID	Cis men	33.7%	11.9%	54.5%	100.0%
		(34)	(12)	(55)	(101)
	Cis women	34.9%	13.2%	51.9%	100.0%
		(138)	(52)	(205)	(395)
	Trans men	33.8%	13.8%	52.3%	100.0%
		(22)	(9)	(34)	(65)
	Trans women	25.7%	25.7%	48.6%	100.0%
		(9)	(9)	(17)	(35)
	Non-binary	27.3%	19.0%	53.7%	100.0%
		(59)	(41)	(116)	(216)
	Intersex	22.7%	18.2%	59.1%	100.0%
		(10)	(8)	(26)	(44)
	Other	35.7%	14.3%	50.0%	100.0%
		(5)	(2)	(7)	(14)
Total		(277)	(133)	(460)	(870)

### Masculine Dominance and Feminine Submission

One aim of this study was to investigate, beyond gender identity, how does gender impact orgasm outcomes? In our quantitative data, as shown in Table 4, we found that having a feminine partner led to an increased likelihood of experiencing an orgasm at the last sexual interaction (significant at the 0.05 level). We did not find comparable significance for masculinity, which may be due in part to the smaller numbers of cis men and trans men in the sample who are most likely to report higher masculinity levels.

**Table 4:** Femininity of partner and report of orgasms at last sexual interaction

	Orgasm	Not sure	No orgasm	Total
Femininity 1—Not feminine	59% (182)	8% (24)	33% (101)	100% (308)
Femininity 2	67% (171)	6% (15)	26% (70)	100% 256
Femininity 3	71% (89)	9% (11)	20% (25)	100% (125)
Femininity 4	72% (94)	5% (6)	22% (29)	100% 130
Femininity 5—very feminine	78% (68)	6% (5)	17% (15)	100% 88
Total	(604)	(61)	(240)	(907)

Chi-squared value 21.227—DF 12

Qualitatively, participants often noted that they took cues on how to perform based on the masculinity or femininity that their partners displayed; therefore, accounting for why femininity, beyond gender identity, influenced expectations and actions in sexual interactions. A good number of respondents assumed a more dominant role if their partners were cis women and a more submissive role with cis men. Others looked for more subtle clues about their partner’s masculinity and femininity to determine their role in the interaction. This is flexibility best demonstrated by River (O):

[I’m] entirely passive/receptive/bottom with masculine partners; usually more of a mix with feminine partners. For GNC [gender non conforming] or non binary partners, it depends on the specifics of the encounter.

Jesse (N) felt there were more unspoken rules in having sex with cis women because they are mistaken to be a cis man rather than non-binary in those interactions:

As (more or less) a cis man, I generally feel more pressure to act dominant/aggressive/active with a partner who is a woman (especially if she is cis). There’s usually an unspoken assumption that I should slip into a more traditionally masculine role with a partner who isn’t queer in some way. With men and non binary people, I feel like it’s more okay to just follow what comes naturally to me, instead of focusing on how I “should” act.

This “unspoken assumption” was found throughout participant responses with individuals who had mixed-gender encounters because they relied on presentation of masculinity and femininity and often drew on heteronormative scripts. In Jesse’s (N) case, interactions with cis women involved more presumption of masculinity because they were falsely categorized as cis. Other respondents focused less on other people’s perception of them. For example, Vivian (CW) describes a process of assessing her partner’s masculinity or femininity before setting any expectations:

With cis men or non binary people who I perceive as more masculine than myself, I expect to be penetrated. With cis women who I perceive as more feminine than myself, I expect to penetrate them with a strap-on.

In Vivian’s (CW) case, gender appears to map onto her assessment of masculinity/femininity as she lumps non-binary and men together as more masculine, but separates women as (likely) more feminine. She also limits sex to penetration and relies on heteronormative expectations to her role and there is no indication of what is more pleasurable.

Taking on a more dominant role with women was often taken for granted, participants provided very little explanation as to why they did not choose submission with feminine partners. Gina (CW), however, indicated that her preference for a dominant role was in part due to her limited experience with women:

My sexual encounters with women or female identifying individuals are far more communicative and reciprocal. I play a more dominant role in my sexual experiences and in fact for many years after I first started to sleep with women, I had a top only mind set. Over the years my experience with sex has changed and I think I attribute that to having more female partners.

Gina negates the homeostasis of sexual scripts in discussing how her interactions changed as her partners became more gender expansive and she gained experience. Still, like many other respondents, Gina initially used the dominant heteronormative script that women should be submissive in their sexual encounters. These accounts show the extent to which binary heteronormative scripts are pervasive. They permeate beyond gender identity, and the assumed roles that follow, but also through other components of gender, gender make-up (Harvey, 2020).

### Negotiating Power and Safety

The negotiation of safety and power dynamics are often covert and implicit elements of a sexual interaction (Holland et al., 1992). Sexual interactions exist in the context of societal norms, where women are often expected to be submissive or subordinate to men. We investigate how gender shapes these negotiations for our respondents.

Even when participants took a “less traditional role” for their gender it appeared the extent to which they “played” that role shifted based upon their partner's gender identity. While Gina (CW) says she plays a more dominant role generally, she also specifies that when she started sleeping with women she was “top only” indicating that submission was far less likely with her women partners. Another interpretation of Gina’s response might be that when people enter into sexual interactions with partners of different genders, they also renegotiate power and safety. Conversely, Virginia (CW) notes that she likes to be submissive but it depends on her level of control in the situation:

With a woman I want to be dominated, I want to be the submissive. With a man I want to be more dominant. I do like to be submissive at times but I want to have more control over the situation.

The clarification that she likes to be submissive with men is conditional upon control which was not specified when talking about women. Several respondents mentioned that the difference between having sex with men and women was tied to their feelings of safety. Usually, safety was bound up in discussions about gender expectations and affirming masculinity. For example, Georgia (CW) said the threat of violence and the need for constant reassurance made sex with men less pleasurable:

I also feel like I can let desire build naturally with a woman while men demand constant reassurance that I find what they’re doing sexy at all times. There is also no threat of violence when making sexual decisions with female partners.

Grace (CW) similarly noted:

Sex with cis men, as a cis woman, feels riddled with expectations. I am always more self-conscious about what I look like and often contort my body to appear slimmer, keep my makeup on, and make sure my lingerie matches. And I am usually scared. With cis women (and I imagine, if I were to have sex with a genderqueer person, though I haven’t yet) I can relax and be myself. I am not afraid of being assaulted or my boundaries being crossed. There is clearer communication.

These feelings were also shared by non-binary respondents including one who simply responded “men scare me.” Sam (N) similarly stated:

With men, as an AFAB [assigned female at birth] person, there has been expectations of how I would act and my role during sex, and that is usually to give the man pleasure, and stroke their egos and assure them they performed well and I enjoyed myself, especially during instances where I did not feel completely safe or comfortable and/or was pressured/forced into sex.

Sex is often used as a site to affirm masculinity (Quinn, 2002), and for some participants that created pressure to adopt normative femininity or at the very least, affirm their cis man partner’s sexual prowess. The threat of violence adds a new dimension as to why respondents reported fewer orgasms with cis men. In these cases, participants felt pressure to meet their partner’s expectations, but there was no mention that cis men wanted to center their pleasure. The absence of fear was associated with having a cis woman or gender queer partner that engaged in open communication and shared decision making.

Risk assessment when engaged in sex with cis men tracks with more recent cultural scripts concerning safety and consent which adds an additional dimension to understanding lack of orgasm and pleasure within consensual sexual acts. Compared to other groups, respondents who were socialized as girls (cis women and AFAB non-binary) reported the influence fear had on their expectations. Their scripts included expectations of femininity within binary relationships, presumptions of men’s sexual aggression, and fear that consenting lines would be crossed. The quantitative data also showed that having a partner with a higher score on the feminine scale was also associated with a greater likelihood to experience an orgasm. These findings are likely related to the fact that there is a significant positive relationship between communication and orgasm at last sexual intercourse.

### Communication

We found a significant positive relationship between communication and orgasm with partners (*r*(874) = 0.19 *p* < 0.001), which increased when cis men were removed from the analysis (*r*(756) = 0.20 *p* < 0.001). This relationship was also reflected in our qualitative findings. Queer relationships with cis women relied more heavily on communicating expectations than in sexual encounters with cis men. Communication is also highlighted for people with trans and non-binary partners, but it was less about pleasure and more about establishing boundaries and respecting their partners' relationships with their bodies. As Jordan (N) put it “I am more cautious with trans and non binary folx because of gender identity related bodily triggers.” Violet (CW) also stated:

Depends on their wants and what they are comfortable with. For example, the last gender queer partner I had did not want me to touch their breasts during sex because they didn’t feel their breasts were part of their sexual identity—they felt their breasts were too feminine. Also, sex with women or gender queer partners feels more like we are both equally leading the encounter and guiding each other. With men, it seems to be that either me or the man leads the experience rather than it being navigated together.

Notably, Vivian (CW) feels that sex with women and gender queer partners is more collaborative than with men. Avery (N) similarly wrote that they rely on more nonverbal cues from cis men and more communication with trans men and non-binary partners:

I am often attracted to masculine of center folks (trans men, cis men, and masc non binary folks). With trans men and non binary folks, sex is mostly affected by making sure that they are comfortable and happy with the boundaries we are setting up together. With cis men, sex is affected by more unspoken things - the way they see my body and the expectations they have about my gender (and thus my “role” in bed). There’s a lot more work to be done around consent and navigating boundaries, which I think is considered “not hot” among cis men

Just as Jesse (N) wrote that cis women have unspoken expectations that emerge from assuming their gender, Avery (N) feels that cis men tend to do the same. Both these respondents suggest that sex is more comfortable when they discuss their preferences and boundaries because there is less pressure to fill a specific role. None of the respondents mentioned the threat of violence when reflecting on their experiences with women or queer partners. Instead, these interactions were marked by explicit communication about expectations and boundaries.

## Discussion

Previous research demonstrates disparities in the frequency of orgasm; women in heterosexual relationships experience orgasm less frequently than men in heterosexual relationships and both women and men in same-gender relationships (Carlson et al., 2016; Frederick et al., 2018). Our data confirm and build on these previous findings in three ways. First, our understanding of the orgasm gap is expanded to include a more gender-diverse selection of partner pairings. Along with including non-binary and intersex persons, we also consider trans man and trans woman identities separately from cis man and cis woman identities to better understand how expectations of men and women differ according to cis/trans experience. We find that partners of cis men and trans women are less likely to report an orgasm than partners of cis women, trans men, non-binary people, and intersex people. Worse outcomes for individuals partnered with cis men and trans women may be due, in part, to phallocentrism. Many respondents equated the presence of a penis with penetrative sex or indicated that sex ended after ejaculation.

Trans women in the sample had orgasms that were only slightly lower than that of cis men despite their experiences of transmisogyny (Muñoz-Laboy et al., 2017; Schilt & Westbrook, 2009). While trans women experience fear of violence and gender invalidation in sexual experiences, they have also perhaps been socialized with sexual privilege. As Ridgeway (2011) argued, the repetitive process of doing gender solidifies our expectations and experiences of gender. Therefore, although trans women will have done gender as a woman for many years, we suspect that being socialized to do masculinity would have solidified the expectation to orgasm in sexual interactions. The small pleasure gap between trans women and cis men may have some relationship to specific pressures of stigma, shame, homophobia, and transmisogyny. More research is needed to further understand the impact of gender identity here.

Scripts theory assumes a relative stability of sexual scripts throughout the life course; as first sexual interaction often happens before transition (Averett et al., 2014; Pires et al., 2014) post-transition sexual outcomes may be impacted by these early experiences. In addition, a large part of a person’s sexual education and sexual socialization also happens before transition. We argue that trans women benefit from the childhood and adolescent gender socialization that they should expect pleasure, if all people we socialized (both in culture and formal sex education) in this way perhaps we would see no or less difference in pleasure outcomes by gender identity.

Partners of cis women were most likely to report orgasm at last intercourse and they were closely followed by partners of trans men. The discrepancy between cis men and cis women outside of heterosexual relationships may highlight that heteronormative sexual scripts contribute to the expectation that cis women do not expect an orgasm in the way that cis men do, even in relationships that deviate from normative cis heterosexual couplings.

Trans men have orgasms in sexual interactions near the same frequency as cis women, which could be attributed to a few things. First, women, non-binary, and trans partners communicate their expectations of sex more readily than do cis men, and most of our pairings were heterosexual in nature. Like the partners of trans men in Brown’s (2010) study, respondents shared that they were mindful of their partner’s bodily triggers and worked to respect their boundaries by refusing to engage with body parts that their partner identified as feminine. While some trans individuals have no issue with their genitalia, others rename their genitalia to align with their gender identity, and some engage in sexual interactions in ways that avoid their genitalia (Anzani et al., 2021; Brown, 2010; Pfeffer, 2008, 2014a, 2014b) which can contribute to varying orgasm frequency among some trans individuals. We also suspect that the trans men’s orgasm frequency is more similar to cis women than cis men in part because sexual debut typically occurs prior to transition. Those socialized as girls and young women often learn to deprioritize their own pleasure during sex (Estep et al., 1977; Shtarkshall et al., 2007; Štulhofer et al., 2010). After transitioning, trans folks both intentionally and unintentionally hold onto aspects of their early socialization, part of what Connell (2010) refers to as “doing transgender,” and for trans women this may include decentering their own pleasure during sex.

Our second contribution is that communication and orgasm have a significant positive correlation and when connected to our qualitative data, we find that communication affects partner satisfaction and disrupts gender expectations. Notably, our connected finding that the partners of trans men, non-binary people, and intersex people are also more likely to report orgasm than those of cis men may suggest that inability to assume gender expectations can also prompt explicit communication to boost sexual pleasure and/or affirm gender in sexual practice, leading to a more mutually satisfying sexual interaction. In this way, communication not only increases orgasm rates but affirms gender identity.

Respondents indicated that cis men and women do not readily communicate with their partners and rely more heavily on scripts. This made sex less pleasurable as respondents felt a need to play a role rather than doing what they enjoy. First, the absence of communication leaves boundaries unknown and increases the threat of sexual violence; however, it might also be the case that the threat of violence makes individuals, mostly women and trans folks, more reticent. Despite all respondents in the qualitative data having sexual partners of different genders, it was mostly cis gender women who reported a feeling of lack of safety in sexual interactions. This warrants the question of how fear of assault contributes to the reliance upon sexual scripts and women’s decreased pleasure levels. Secondly, when people assume their partner’s gender and rely on dominant scripts, both parties might fall into roles that are not productive of pleasure or satisfaction. As shown in our findings, Jesse did not like being dominant but felt that cis women expected it and Grace may not have enjoyed what was happening but was too scared to express her needs. Where change/renegotiation is happening, perhaps it is the cultural influence of active decision making around sex and explicit communication around consent and sexual interactions that is pushing this change (Harvey, 2020; Spišák, 2017).

New ways of engaging in sex often prove to be rooted in heteronormative expectations of what men and women do during sex. Our findings are closely aligned with Brown (2010) wherein trans men tended to focus solely on penetrative sex, did not like their partners to initiate sex, and wanted their partners to accentuate their femininity by wearing lingerie. Self-identified bisexual women partnered with trans men easily adjusted to new gender expectations because they drew on their experiences dating cis men. Other partners were triggered by their partner’s transition due to their history of sexual assault. This indicates that just as scripts are determined by one’s own gender and sexual history, people also select interpersonal scripts according to their partner’s gender.

Our third contribution is that we can see how heteronormative roles can persist even outside of cis man/cis woman dyads. Although cis partners relied most heavily upon heteronormative scripts and gender expectations, binary roles of top/bottom, masculine/feminine, dominant/submissive, active/passive, and assertive/receptive are reproduced in same-gender relationships and relationships with trans and non-binary partners. Previous studies have argued that Queer and LBQ folks criticize and subvert heteronormative scripts as they find them to be highly gendered and constraining (Lamont, 2017). Despite this, we find many replicate in their language on gendered expectations. Interestingly, the gender roles a person takes do not appear to be fixed, respondents often determine their roles by assessing their partner’s masculinity and femininity in relation to their own. These roles may offer a sexual script that facilitates a sexual encounter by setting expectations for both partners. This affirms our larger finding that gender is both interactional and relational, as well as illustrating the fluidity of gender in individual actors.

Heteronormative roles and scripts may not be readily applicable or desired for many individuals. Participants mentioned the need for clear communication around desires and activities when gender roles were more ambiguous. Participants also discussed resisting gendered expectations that may be uncomfortable or invalidating for trans and non-binary sexual partners who highlight that orgasm and pleasure are not synonymous. Sexual pleasure can be conceived of and experienced in diverse ways (Thomas & Copulsky, 2021). Gendered differences in orgasm may at times be a result, not of neglect for pleasure, but of specific attention to individual desire and preference as communicated by one’s partner. In these circumstances, expected sexual scripts may be an obstacle, but renegotiating these scripts may be a productive path toward sexual pleasure.

Broadly, our findings show that gender is not only interactional but relational and intimate relations are shaped by heterosexual scripts. Heteronormative scripts are so deeply embedded in our culture and socialization processes that they are often enacted unconsciously, despite conscious resistance to them. By analyzing the data from a new angle, considering categories based on partner gender (e.g., all people who have sex with cis men) rather than individuals’ own gender and orientation (e.g., separating heterosexual women from gay men), we come to understand the deep ways heteronormative scripts impact our sexual lives beyond heterosexual couplings. Our qualitative data also lend an additional lens to our consideration, allowing participants to describe how gender impacts sexuality. At times respondents conflated sex and gender which demonstrated cisnormative ideas about how genitals translate to sexual roles. As others have documented, despite issues with essentialist understandings of gender, sex, and genitalia, it is often compelling to fall back on “biologically” based scripts and interpretations of social conventions (Bridges, 2010; Epstein, 1988). Other times, respondents viewed gender and sex as distinct and operated with regard to their partner’s preferences. Thus, sexual scripts may be based on the assumed gender roles related to physical anatomy and/or social gender presentation. As discussed in doing gender, people heavily rely on their own understandings of masculinity, femininity, and sexuality to inform how they interact in social interactions, this remains true in sexual interactions.

However, we found that sexual behavior is not based on being held accountable to one’s own gender identity, rather, behavior was primarily determined by assessing one’s masculinity and femininity in relation to that of their sexual partners. This is where the value of gender make-up (Harvey, 2020) comes in, as we are able to assess the impact of gender based upon a more rounded measure of social gender components that might be used to navigate gendered interactions. As our results show, even those with gender-diverse sexual histories reply upon heteronormative scripts. heterosexuality may not be mandated, but heteronormativity remains compulsive in determining roles. When we asked our participants about gender, they described roles and genitalia, indicating that binary conceptions of gender permeate how people approach sexual interactions.

Although we see the pervasiveness of heteronormative expectations, especially for cis/hetero sexual interactions, we do not understand why people view gender roles as oppositional in sexual interactions. Are socialization messages so strong or impactful in adolescents that people well into their 20’s and 30’s are unable to act outside of dominant scripts? Further research into the diverse pleasure experiences of these populations and sexual interactions is needed to parse out the relationship between gender, sexual partners, and sexual pleasure.

### Limitations

In juxtaposing the quantitative and qualitative findings here, it is important to highlight again that only participants who previously had sex with partners of more than one gender answered the qualitative questions. While the themes we identified do speak to parallels in the quantitative findings, the patterns here for participants who have sex with partners of more than one gender do not necessarily represent participants who only have sex with partners of one gender.

We also acknowledge that this sample is not random, as participants were recruited through online and snowball sampling, intentionally over-sampling those with minority gender/sex identities. This limits broader assumptions about these findings being representative. For example, women often disproportionately volunteer for survey research (Smith, 2008); if this pattern is a result of gender socialization, we might also expect that non-binary people who are AFAB volunteer disproportionately compared to non-binary people who are AMAB (assigned male at birth). However, it is difficult to distinguish this in the data from a possibility that a higher proportion of AFAB individuals identify as non-binary.

Our interpretation of the participant gender and sex/marker questions may not match participants’ intent and identities. For example, we categorized individuals who responded both that their gender was woman and sex was female (and did not select a “trans” option) as cis women. However, it is also possible that some trans individuals answered these questions the same way. Participants may think of being trans as a part of their history rather than part of their current identity, as this pair of questions is framed in the present tense. This might be the case particularly for participants who have socially and medically transitioned. Future studies might approach these questions differently, such as including a question that directly asks “are you transgender?”

We face similar challenges in considering participants’ partners’ genders, which we categorized based on a single question about gender identity. Again, participants may choose to answer this question based on current gender identity without disclosing a transgender history. Participants may also not know that a partner is trans.

As this paper highlights the importance that both genitalia and socialization may also play in outcomes for partnered sex, a future study might benefit from including multiple questions about partner’s current gender identity, sex/markers, and gender/sex assignment. For example, experiences may vary between non-binary partners who are AFAB and AMAB, a distinction our questions do not capture. We also propose the further exploration of how gender expectations and roles can change with the same partners as a small number of participants discussed switching roles with the same partner based on the fluid experiences of their own or their partner’s gender.

### Conclusion

This paper aimed to address areas neglected by previous studies on gender disparities in sexual pleasure by focusing both on the experiences of transgender, non-binary, and intersex people and on the experiences of people who have had partners of more than one gender. With this focus, we have considered how gender is experienced relationally. Our findings show that accountability to one’s own gender does not solely determine how one engages in sexual interactions. Sexual behavior is also determined by the gender/sex and gender presentation of their partners. These private, intimate experiences are also critically impacted by social scripts and norms, with roles and expectations often shaped from much earlier in life.

Gendered and heteronormative scripts do still play a part in sexual partnerships that include transgender, non-binary, and intersex individuals. However, when scripts are less readily applicable, partners turn more to explicit communication as they seek sexual pleasure and navigate their roles, behaviors, and desired outcomes. Education and advocacy work focused on sexual communication should include not only navigating safer sex, but also consideration of roles, desire, and pleasure. This focus may benefit partnerships with same-gender, transgender, and non-binary partners, where existing scripts are less readily applicable, but may also benefit partnerships between cis men and cis women, where an unquestioned reliance on existing scripts could also be disadvantageous.

## Appendix 1

### Gender Make-up Scale

As the model shows, masculinities and femininities are measured across four components of gender to form a composite of a person's gender make-up: identification, physical expression, interactional expression, and interests.

## Footnotes

1 Respondents listed first.
